# 

**Published:** 1840-05

**Authors:** 


### DENTAL SCIENCE,

### FOR MAY, 1840.

Vol I.—No. V.

EDITED BY

CHAPIN A. HARRIS, M. D.

Baltimore.

ELEAZAR PARMLY,

New-York.

PUBLISHING COMMITTEE,

E. PARMLY, | E. BAKER,

S. BROWN.

NEW YORK,

GEORGE ADLARD, 168 BROADWAY,

BALTIMORE,

ARMSTRONG & BERRY.

PRICE---THREE DOLLARS PER ANNUM.

J. C. Kelley, Printer.

